# Remote monitoring contributes to preventing overwork-related events in health workers on the COVID-19 frontlines

**DOI:** 10.1093/pcmedi/pbaa014

**Published:** 2020-05-11

**Authors:** Faming Zhang, Huiquan Wang, Ruijuan Chen, Wenzhi Hu, Yuexia Zhong, Xin Wang

**Affiliations:** Medical Center for Digestive Diseases, Second Affiliated Hospital of Nanjing Medical University, Nanjing 210011, China; Biomedical Engineering Department, School of Life Sciences, Tiangong University, Tianjin 300387, China; Biomedical Engineering Department, School of Life Sciences, Tiangong University, Tianjin 300387, China; Department of Cardiology, Second Affiliated Hospital of Nanjing Medical University, Nanjing 210011, China; Wuhan Huoshenshan Hospital, Wuhan, China; Tangdu Hospital, Fourth Military Medical University, Xi'an 710038, China; Wuhan Huoshenshan Hospital, Wuhan, China; Tangdu Hospital, Fourth Military Medical University, Xi'an 710038, China

**Keywords:** coronavirus, COVID-19, overwork, sudden death, wearable device, 5G

## Abstract

Fighting on the frontlines against the coronavirus disease 2019 (COVID-19) pandemic, health workers are at high risk of virus infection and overwork-related sudden death and disorders including cardiovascular diseases and stress. When we noted the increase of overwork-related sudden deaths in physicians and nurses in the first 2 weeks after lockdown of Wuhan, we organized the ‘Touching Your Heart’ program by remote monitoring, aiming to protect health workers from overwork-related disorders through integrated volunteer work by physicians and medical engineering researchers from Wuhan Huoshenshan Hospital, Nanjing Medical University, and Tiangong University. By remotely monitoring the health conditions of the medical aid team working at Wuhan Huoshenshan Hospital, the program successfully helped in avoiding severe overwork-related events. The results from our program should be used to remind frontline health workers around the world to take precautions against overworked-related severe events, and show that precision monitoring is effective in improving work efficiency and maintaining a sustainable workforce during emergency situations like a pandemic.

## High risk of overwork-related events during COVID-19 pandemic

The coronavirus disease 2019 (COVID-19), which started in December 2019 and quickly spread around the world, had more than 2 200 000 confirmed cases up to 18 April 2020. Physicians, nurses, and other health workers are on the frontlines of the pandemic, working under great tension and pressure, committing their lives to controlling and preventing the spread of the disease. In China for example, since the lockdown of Wuhan on 23 January 2020, most hospitals and health workers have been in an emergency work mode. The Chinese government organized over 42 000 physicians, nurses, and medical technicians outside Hubei to work with 500 000 local health professionals in the province.^1^ Among them, 1415 persons including physicians, nurses, and other staff worked at the Wuhan Huoshenshan Hospital with 1000 beds, which was built within 10 days of the lockdown of Wuhan city, mainly for treating severe and critical COVID-19 cases.

Because of a lack of health workers and personal protective equipment (PPE), most of the doctors and nurses have to work for a long time while wearing protective clothes and appliances. Hypoxia, sweat loss, insufficient energy, and inconvenience to go to the toilet are the main problems when working with sealed protective appliances in the ward. They are therefore at high risk of overwork-related sudden death and disorders including cardiovascular diseases.

Besides the physical conditions resulting from heavy work burden, the fear and panic caused by the outbreak itself and the control measures is another problem facing medical staff.^2,3^ The psychological stress adds to the physical fatigue and, together, these may result in serious health events in health workers. While the pandemic in China has been successfully controlled, we were sorry to lose 18 health workers to overwork-related events, which took place 24.89 ± 3.67 days (range 4–56 days) after the lockdown, in workers of average age 47.17 ± 2.54 (range 28–69) years old.

## The ‘Touching Your Heart’ program for frontline physicians and nurses

In response to the increase of overwork-related sudden deaths in the first 2 weeks after lockdown of Wuhan City, we devised a new technological method using remote monitoring for early diagnosis of severe heart events in researchers and physicians managing COVID-19 patients.

**Figure 1.**
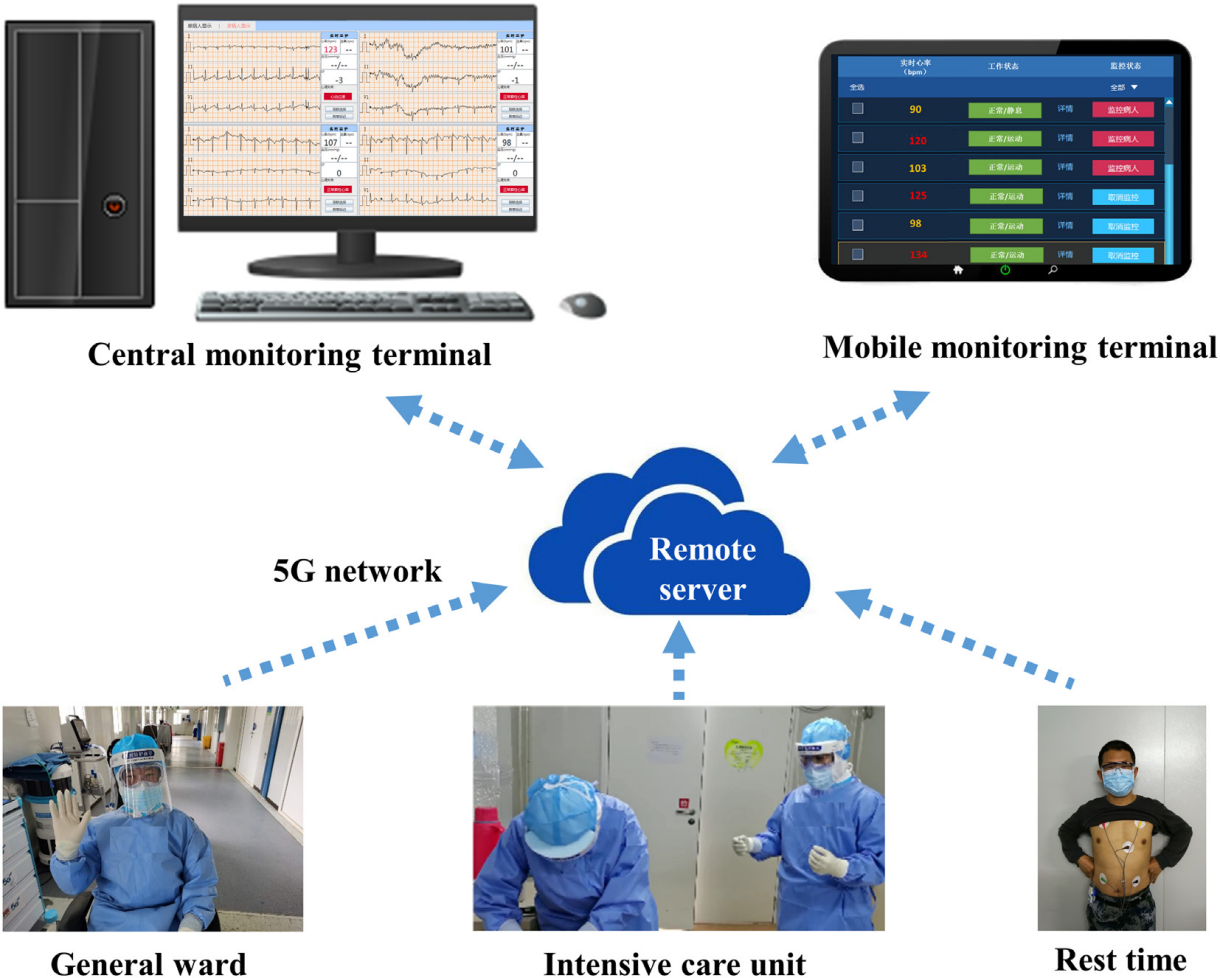
Schematic drawing illustrating the ‘Touching Your Heart’ program.

We organized the ‘Touching Your Heart’ program, integrating physicians and medical engineering researchers from universities as a volunteer team, which aimed to protect health workers from overwork-related disorders. The cooperation involved Xin Wang's medical aid team at Huoshenshan Hospital in Wuhan, Faming Zhang's physician team at the Second Affiliated Hospital of Nanjing Medical University in Nanjing, and Huiquan Wang's engineering team at Tiangong University in Tianjin. This monitoring program was approved by the ethical committee at Wuhan Huoshenshan Hospital. All participants were voluntary, informed and agreed to cooperate in the whole process. A set of wearable devices and online fatigue surveys were developed to remotely monitor the physical condition of the health workers via the 5G network, which refers to the fifth generation of wireless transmission technology (Fig. 1). The 5G network has shown great advantages in hospital intelligence services, including allowing automatic patient monitoring via wearable devices carried by patients.^4^ The wearable monitoring devices in our program are not hampered by the protective clothing, sweat, or work performance of the physicians and nurses.

## Remote monitoring warning against overwork in health workers

The remote monitoring integrated multi-source information including an electrocardiogram, work load, work time, and fatigue level, to warn against overwork-related cardiac events. The design was that the monitoring system could identify those physicians or nurses who were overworked and should be exchanged out for rest or further treatment. Altogether we collected 153 monitoring times for physicians and nurses at work and in rest. Myocardial ischemia complicated arrhythmia was observed in a physician in his 50s. Subsequent coronary computer tomography arteriography showed more than 80% coronary artery stenosis. One nurse was monitored to have frequent ventricular premature beats. They were required to quit work for treatment. Arrhythmia was found 40 times in the aid team.

**Figure 2.**
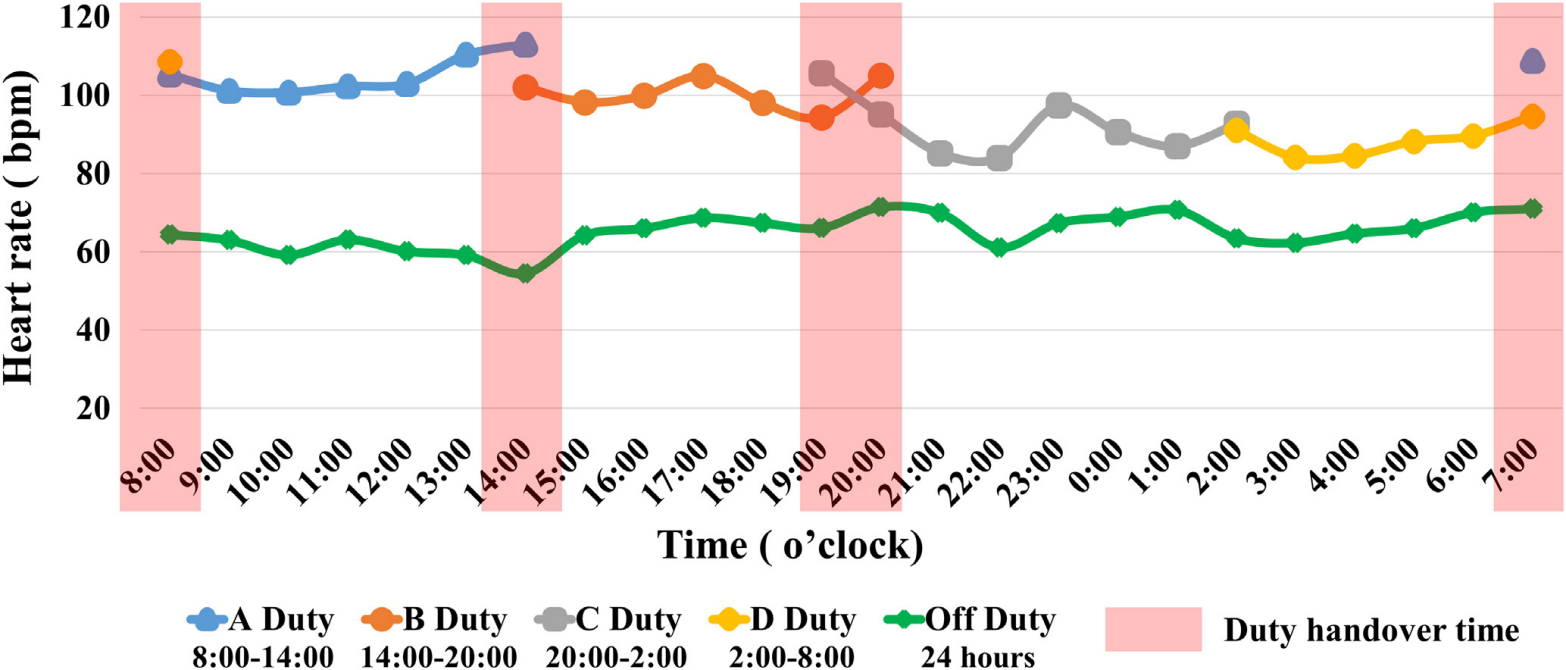
Heart rate changes of nurses and physicians caring for COVID-19 patients.

We observed that the heart rate of physicians and nurses peaked at 8:00–14:00 (106 ± 17.45 bpm; Fig. 2). The work schedule and service force were updated by nursing division based on the present integrated analysis, which might contribute to improving the work efficiency and reducing the overwork-related serious events for nurses. By doing so, the Touching Your Heart program contributed to preventing overwork-related severe events in the medical aid team.

## Conclusions

With the development of pandemic, the soaring confirmed and suspected cases present an enormous workload for health workers, policemen, and other staff on the frontlines globally, who will have to face such challenges for at least a few more months. To fight the pandemic, we need to have sustainable workforce in these teams. Our ‘Touching Your Heart’ program was a pilot for taking care of the health of the workforce through remote monitoring. Such precision monitoring shows promise in improving work efficiency and maintaining a sustainable workforce during times of emergency like a pandemic.
